# Primary amelanotic leptomeningeal melanocytosis mimicking subarachnoid hemorrhage: a case report of the deceptive masquerader

**DOI:** 10.3389/fonc.2026.1768860

**Published:** 2026-04-30

**Authors:** Yu-Chung Juan, John S. Kuo, Chun-Chung Chen, Ren-Ching Wang, Der-Yang Cho, Yu-Kai Cheng

**Affiliations:** 1 Department of Neurosurgery, China Medical University Hospital, Taichung, Taiwan; 2 Neuroscience and Brain Disease Center, China Medical University, Taichung, Taiwan; 3 Graduate Institute of Biomedical Sciences, China Medical University, Taichung, Taiwan; 4 Department of Neurosurgery, Dell Medical School, The University of Texas at Austin, Austin, TX, United States; 5 Graduate Institute of Acupuncture Science, China Medical University, Taichung, Taiwan; 6 Department of Pathology, China Medical University Hospital, Taichung, Taiwan; 7 China Medical University Children’s Hospital, Taichung, Taiwan

**Keywords:** amelanotic, angiogram-negative hemorrhage, CNS neoplasm, diagnostic mimic, immunohistochemistry, primary diffuse leptomeningeal melanocytosis, subarachnoid hemorrhage

## Abstract

**Background:**

Primary diffuse leptomeningeal melanocytosis (PDLM) is a rare central nervous system neoplasm. Diagnosis is challenging in amelanotic variants because they lack the characteristic T1 hyperintensity on magnetic resonance imaging (MRI), leading to confusion with infectious meningitis or hemorrhage.

**Case Presentation:**

A 24-year-old male presented with severe headache and neck stiffness. Initial computed tomography suggested a subarachnoid hemorrhage (SAH); however, digital subtraction angiography was negative. MRI revealed diffuse leptomeningeal enhancement without T1 hyperintensity. Cerebrospinal fluid (CSF) analysis was non-diagnostic. Owing to progressive clinical deterioration despite CSF diversion, a biopsy was performed. Histopathology confirmed amelanotic PDLM with positive S-100, HMB-45, and Melan-A markers, but negative Fontana-Masson staining. Hematology-oncology consultation recommended MEK inhibitor therapy based on MAPK pathway rationale; however, molecular genetic profiling was pending and the patient’s rapid neurological deterioration precluded initiation of targeted therapy. The patient succumbed to the disease approximately 3 months after biopsy.

**Conclusion:**

This case highlights the diagnostic pitfalls of amelanotic leptomeningeal melanocytosis and the narrow therapeutic window in advanced PDLM. In young patients with “angiogram-negative SAH” or unexplained leptomeningeal enhancement, the absence of T1 hyperintensity should not exclude melanocytic neoplasms. Early biopsy and prompt molecular profiling are crucial for timely diagnosis and treatment planning.

## 1 Introduction

Primary diffuse leptomeningeal melanocytosis (PDLM) is a rare neoplasm arising from neural crest-derived melanocytes located within the leptomeninges, predominantly at the base of the brain and upper cervical spinal cord. These neoplasms range from circumscribed melanocytomas to diffuse melanocytosis and malignant melanomatosis. Although melanin-rich variants typically exhibit intrinsic T1-weighted hyperintensity on magnetic resonance imaging (MRI) due to the paramagnetic properties of melanin, the amelanotic variant represents a distinct diagnostic challenge. Because they lack this pathognomonic signal, amelanotic lesions often masquerade as more common entities, such as infectious meningitis, neurosarcoidosis, or subarachnoid hemorrhage (SAH). Notably, PDLM can occur in adults without neurocutaneous stigmata such as large congenital melanocytic nevi, further obscuring clinical suspicion (1).Herein, we present a case of primary amelanotic leptomeningeal melanocytosis in a young adult. The patient’s presentation mimicked SAH on computed tomography (CT) and inflammatory meningitis on MRI, leading to a complex diagnostic course. This report emphasizes the critical importance of recognizing “angiogram-negative hemorrhage” as a potential mimic of high-cellularity neoplasms and details the histopathological markers required to resolve this dilemma. To our knowledge, the concurrent presentation of CT pseudo-hemorrhage appearance and complete absence of T1 hyperintensity in the same amelanotic PDLM patient—leading to a multi-step diagnostic odyssey spanning nine months—has been rarely documented in the literature, underscoring the clinical significance of this case.

## 2 Case description

A previously healthy 24 year-old man presented with a two-week history of severe headaches, nausea, vomiting, and neck stiffness. The patient had no history of cutaneous melanoma or other systemic malignancies. On admission, neurological and general physical examinations were nonfocal.

**Figure 1:**
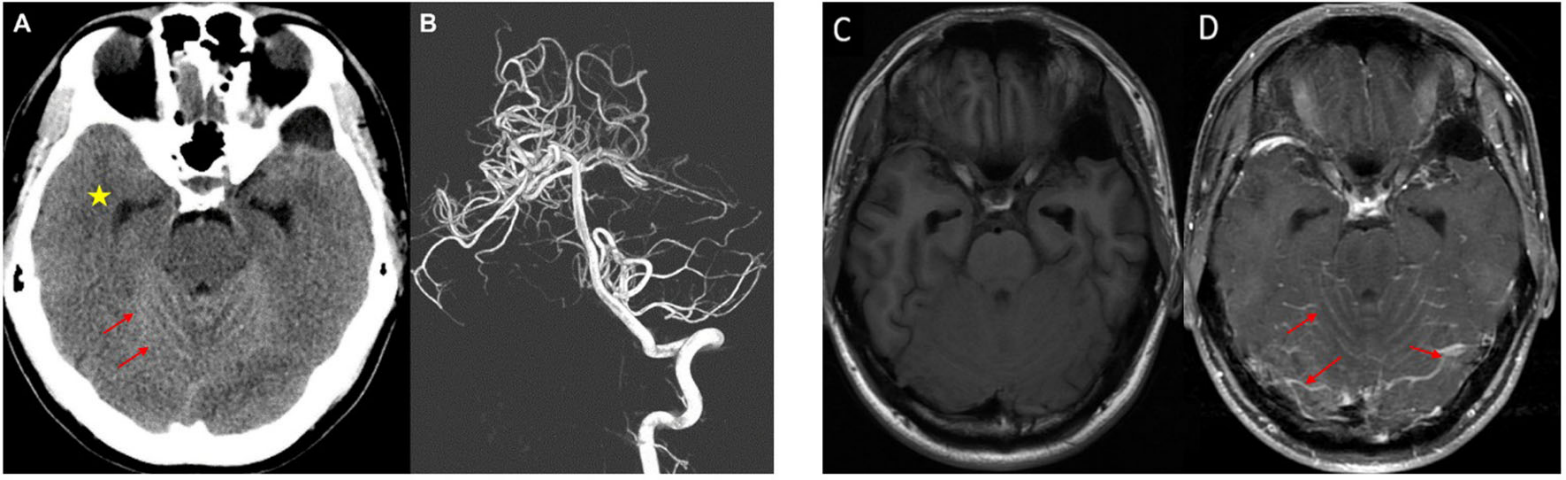
**(A)** Non-contrast head CT demonstrating diffuse hyperattenuation within the posterior fossa sulci (red arrows), mimicking subarachnoid hemorrhage. Note the dilated temporal horn of the lateral ventricle (yellow star), indicating early obstructive hydrocephalus. **(B)** Digital subtraction angiography showing no evidence of aneurysm or vascular malformation. **(C)** Pre-contrast T1-weighted MRI at the posterior fossa level demonstrating no abnormal leptomeningeal signal hyperintensity, a finding atypical for melanin-containing melanocytic lesions and representing the key diagnostic pitfall of this amelanotic variant. **(D)** Post-contrast T1-weighted MRI at the same level revealing diffuse leptomeningeal enhancement along the posterior fossa sulci and cerebellar folia (red arrows), consistent with extensive melanocytic infiltration.

Initial non-contrast head CT (GE Revolution EVO scanner (GE Healthcare, Chicago, Illinois, USA) revealed diffuse hyperattenuation within the posterior fossa sulci and ambient cistern (red arrows), with associated dilatation of the temporal horn of the lateral ventricle (yellow star) suggesting early obstructive hydrocephalus, the which was radiologically interpreted as a SAH (**Figure 1A**). Consequently, the patient underwent CT angiography and digital subtraction angiography. Both studies were negative for aneurysms or other vascular malformations (**Figure 1B**). Pre-contrast T1-weighted MRI at the posterior fossa level demonstrated no abnormal leptomeningeal signal hyperintensity (**Figure 1C**), a finding atypical for melanin-containing melanocytic lesions and representing the key diagnostic pitfall of this amelanotic variant. Post-contrast T1-weighted MRI at the same level revealed diffuse leptomeningeal enhancement along the posterior fossa sulci and cerebellar folia (red arrows, **Figure 1D**), consistent with extensive leptomeningeal infiltration. Due to the development of acute hydrocephalus on day 4 of admission, emergent external ventricular drain (EVD) insertion via right Kocher’s point was performed for urgent intracranial pressure management. CSF analysis revealed clear fluid with normal glucose and protein levels, and no pleocytosis. Cytological examination was negative for malignant cells. Infectious workup included CSF cultures for bacteria, tuberculosis, fungi, and acid-fast bacilli (AFB), cryptococcal antigen testing, enterovirus culture, and PCR for herpes simplex virus (HSV), Epstein-Barr virus (EBV), and cytomegalovirus (CMV). All results were negative. Following clinical stabilization, the EVD was converted to a permanent right ventriculoperitoneal (VP) shunt on day 10, and the patient was discharged on day 15.

The patient was readmitted approximately 4 weeks after initial discharge due to persistent symptoms. Further workup was performed in consultation with the rheumatology department, including serum ANCA, cryoglobulin, ENA panel (anti-dsDNA, SS-A, SS-B, Sm, RNP, and anti-ribosomal P antibody), immunoglobulins (IgG/A/M), complement levels (C3/C4), and CSF IgG index. Gallium scintigraphy was also performed to exclude systemic inflammatory disease. All results were negative or within normal limits.

After multiple subsequent readmissions due to persistent symptoms and refractory intracranial hypertension, and progressive clinical deterioration with follow-up brain MRI showing worsening leptomeningeal enhancement, a right fronto-temporal decompressive craniectomy and open biopsy was performed approximately 9 months after initial presentation (**Supplementary Figure 1**.).

**Figure 2:**
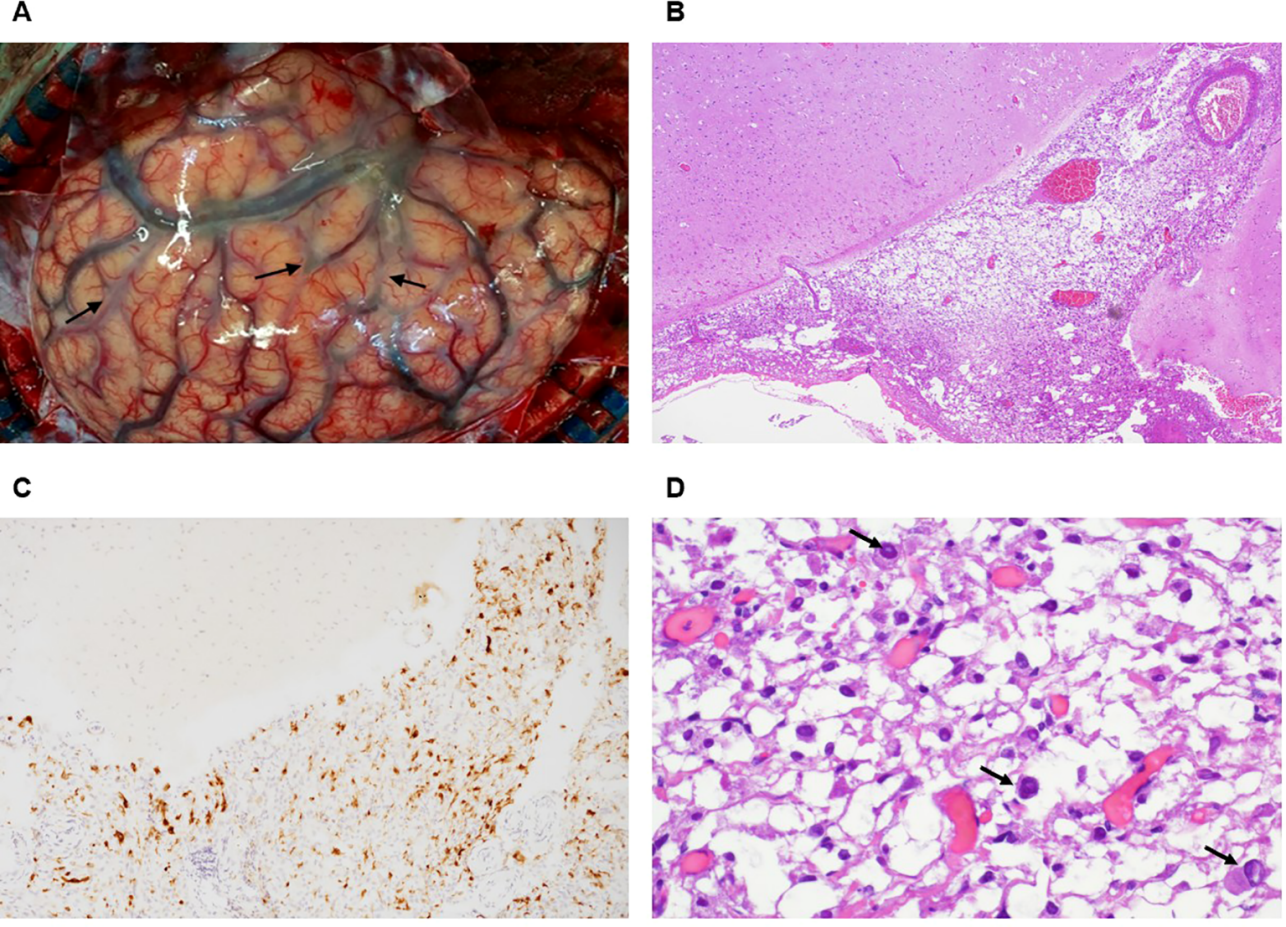
**(A)** Intraoperative photograph demonstrating diffuse cerebral swelling and congestion with grayish discoloration of the arachnoid membrane (arrows), indicating diffuse leptomeningeal infiltration. **(B)** H&E staining (40x) demonstrating clear cell infiltration aggregating in the leptomeningeal space (Virchow-Robin spaces). **(C)** Immunohistochemical staining for Melan-A (200x) demonstrating cytoplasmic positivity in tumor cells, confirming melanocytic origin. **(D)** H&E staining (400x) showing focal nuclear atypia with intranuclear inclusions (arrows).

Intraoperative findings revealed diffuse cerebral swelling and congestion (**Figure 2A**). Histopathologically, clear cell infiltration was observed aggregating in the Virchow-Robin spaces; many of these cells exhibited intranuclear inclusions consistent with a melanocytic neoplasm (2). Immunohistochemical staining was essential for diagnosis, and the tumor cells exhibited strong immunoreactivity for Melan-A (**Figure 2C**), S-100, and HMB-45,as documented in the original pathology report. Fontana-Masson staining was also performed and confirmed the absence of melanin granules, establishing the diagnosis of the amelanotic variant of leptomeningeal melanocytosis (**Figures 2B, D**). Representative photomicrographs for S-100, HMB-45, and Fontana-Masson staining are unavailable due to the retrospective nature of this case; results are based on the original pathology report documentation.

Following the diagnosis, hematology-oncology consultation was obtained. MEK inhibitor therapy was recommended based on literature suggesting MAPK pathway activation in CNS melanocytic neoplasms. However, molecular genetic profiling (KRAS/NRAS/RAF) was still pending at the time of consultation, and the patient’s rapid neurological deterioration precluded initiation of targeted therapy. The patient succumbed to the disease approximately 3 months after biopsy.

## 3 Discussion

This case highlights the deceptive radiological appearance of leptomeningeal melanocytosis. Typically, melanomas are hyperintense on T1-weighted MRI sequences and hypointense on T2-weighted sequences owing to the paramagnetic effects of stable free radicals within melanin. In amelanotic variants, this “diagnostic beacon” is lost. As shown in **Figure 1C**, the patient’s lesion was isointense and indistinguishable from non-specific meningitis or carcinomatosis.

Furthermore, the initial CT findings of hyperattenuation mimicked those of SAH. This “pseudo-hemorrhage” appearance was likely due to the high cellular density of the neoplastic infiltration within the subarachnoid space. It is important for clinicians to recognize that hyperattenuation on CT without angiographic evidence of vascular pathology should prompt immediate consideration of high-cellularity tumors (such as lymphoma or melanocytosis) rather than only focusing on occult vascular causes.

The combination of meningeal enhancement and negative vascular studies creates a broad differential diagnosis. **Table 1** summarizes the key features distinguishing amelanotic primary diffuse leptomeningeal melanocytosis (PDLM) from its mimics (3, 4).

**Table 1:** Differential diagnosis of diffuse leptomeningeal enhancement based on imaging.

Feature	Amelanotic PDLM (Current Case)	Subarachnoid Hemorrhage (SAH)	Infectious Meningitis	Leptomeningeal Carcinomatosis
CT Appearance	Hyperdense (mimics blood due to hypercellularity)	Hyperdense (blood in subarachnoid space)	Normal or mild edema (rarely hyperdense)	Normal or isodense
MRI T1 (Pre-contrast)	Isointense / Hypointense (no melanin signal)	Variable (depends on age of bleed)	Isointense	Isointense
MRI T1 (Post-contrast)	Diffuse leptomeningeal enhancement	Minimal / no enhancement	Diffuse / basal enhancement	Nodular or diffuse enhancement
Angiography	Negative	Aneurysm / AVM often positive	Negative (vasospasm possible)	Negative
CSF Findings	Negative cytology (often false negative); normal / high protein	Xanthochromia; red blood cells	High WBC; low glucose; high protein	Positive cytology (malignant cells)

AVM, arteriovenous malformation; CSF, cerebrospinal fluid; CT, computed tomography; MRI, magnetic resonance imaging; PDLM, primary diffuse leptomeningeal melanocytosis; SAH, subarachnoid hemorrhage; WBC, white blood cell.

As illustrated, amelanotic leptomeningeal melanocytosis shares similar CT features with SAH as well as similar MRI features with meningitis, creating a “perfect storm” for misdiagnosis.

Compared with previously reported cases of PDLM, the present case is notable in several respects. First, the simultaneous presence of CT pseudo-hemorrhage and complete absence of T1 hyperintensity—representing dual imaging mimicry—has rarely been described in combination within a single case. Most prior reports of amelanotic PDLM describe either CT hyperdensity or atypical MRI findings, but seldom both. Second, the patient’s young age (24 yo) and absence of neurocutaneous stigmata such as melanocytic nevi or Ota’s nevus made clinical suspicion particularly challenging. Third, completely negative CSF cytology despite extensive leptomeningeal involvement underscores the well-recognized but underappreciated false-negative rate of CSF cytology in PDLM, which has been reported to exceed 50% in diffuse variants (5–7). Finally, the nine-month diagnostic odyssey over multiple hospitalizations highlights the cumulative burden of diagnostic delay inherent to PDLM. This protracted course reflects not a failure of clinical judgment, but rather the fundamental diagnostic challenge posed by this rare entity. A comprehensive literature review of 26 PDLM cases identified unspecific clinical presentation — including unrecognized intracranial hypertension — as the most frequently cited reason for diagnostic delay, occurring in 67% of reported cases (8). In our patient, the initial CT appearance mimicked SAH, prompting cerebrovascular investigation; subsequent MRI and CSF findings raised suspicion for inflammatory or infectious meningitis, necessitating extensive rheumatological evaluation. Only after exhaustion of all non-invasive diagnostic modalities across four hospitalizations was surgical biopsy performed. This case strongly supports the emerging consensus that early tissue biopsy should be considered when unexplained progressive leptomeningeal enhancement persists despite inconclusive non-invasive workup (9).

Histopathology is the gold standard for diagnosis. Clear cell infiltration within the Virchow-Robin space is a characteristic feature. In the absence of melanin pigment (confirmed by negative Fontana-Masson staining), the panels of S-100, HMB-45, and Melan-A are non-negotiable for diagnosis. Although Fontana-Masson negative staining defines the “amelanotic” nature, this should not lead pathologists away from a melanocytic diagnosis if the immunohistochemistry (IHC) panel is positive (2, 10, 11).

From a molecular perspective, primary central nervous system (CNS) melanocytic neoplasms often harbor mutations in the MAPK pathway, distinct from cutaneous melanomas. While the cutaneous types exhibit frequent BRAF mutations, CNS variants (similar to uveal melanoma) more commonly carry mutations in GNAQ, GNA11, and NRAS (12). This provides a rationale for the use of MEK (such as trametinib or binimetinib) or PKC inhibitors. In the present case, hematology-oncology consultation recommended MEK inhibitor therapy; however, molecular genetic profiling was pending and the patient’s rapid neurological deterioration precluded the initiation of targeted therapy. This inability to commence treatment despite a clear molecular rationale further highlights the urgent need for genomic profiling (next-generation sequencing) at the time of biopsy to guide targeted therapy; however, standardized protocols are yet to be established (13–15).

## 4 Conclusion

Primary amelanotic leptomeningeal melanocytosis is rare, aggressive, and presents several diagnostic challenges. It mimics SAH on CT and inflammatory meningitis on MRI. This case experience underscores that, in young patients with unexplained leptomeningeal enhancement and negative vascular imaging, the absence of T1 hyperintensity does not exclude melanocytic neoplasms. We advocate for incorporating amelanotic melanocytic neoplasms into the differential diagnosis of angiogram-negative CT hyperdensity. Early tissue biopsy with a comprehensive melanocytic IHC panel—including Melan-A, HMB-45, S-100, and Fontana-Masson staining—should be performed regardless of the presence or absence of T1 hyperintensity. Furthermore, this case underscores the critical importance of simultaneous genomic profiling at the time of biopsy. Prompt diagnosis via molecular markers is crucial for targeted therapy due to the narrow therapeutic window in advanced PDLM.

## 5 Patient perspective

The patient and his family were initially relieved when a ruptured aneurysm was not diagnosed on angiography. However, the subsequent weeks of uncertainty with negative spinal taps and worsening pain caused significant distress. When the diagnosis of a rare malignancy was confirmed, the patient’s rapid progression left little time for psychological adjustments. The family expressed that earlier identification, which likely would not change the ultimate outcome, would have provided them more time for palliative planning.

## Data Availability

The datasets presented in this article are not readily available because of ethical and privacy restrictions. Requests to access the datasets should be directed to the corresponding author/s.

## Supplementary material

Supplementary Figure 1 Clinical timeline of the diagnostic and therapeutic course, depicting the sequence of key events from initial presentation to biopsy and final outcome.
