# Supplementary figures and images for: Primary amelanotic leptomeningeal melanocytosis mimicking subarachnoid hemorrhage: a case report of the deceptive masquerader
Source: Front Oncol. 2026 Apr 30;16:1768860. doi: 10.3389/fonc.2026.1768860 (PMC13171300; doi:10.3389/fonc.2026.1768860)

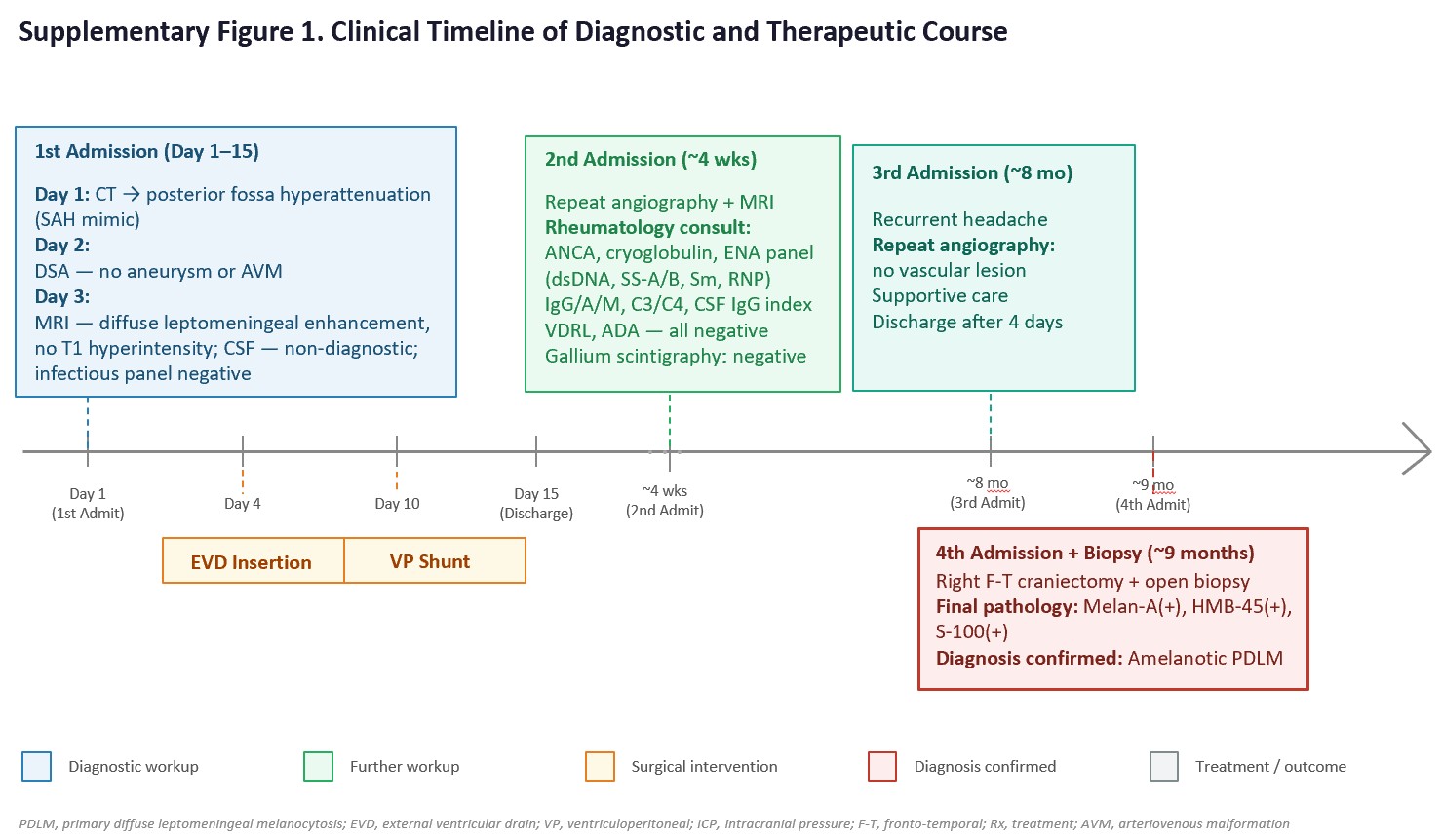

Supplement: Supplementary Figure 1 — Clinical timeline of the diagnostic and therapeutic course, depicting the sequence of key events from initial presentation to biopsy and final outcome. [file Image1.jpeg]
